# Safewards: Changes in conflict, containment, and violence prevention climate during implementation

**DOI:** 10.1111/inm.12762

**Published:** 2020-07-20

**Authors:** Geoffrey L. Dickens, Tracy Tabvuma, Steven A. Frost, SWSLHD Safewards Steering Group

**Affiliations:** ^1^ Department of Nursing, Midwifery and Health Faculty of Health and Life Sciences Northumbria University Newcastle UK; ^2^ South Western Sydney Local Health District Warwick Farm New South Wales Australia; ^3^ Centre for Applied Nursing Research Ingham Institute for Applied Medical Research Liverpool New South Wales Australia; ^4^ School of Nursing and Midwifery Western Sydney University Sydney New South Wales Australia

**Keywords:** aggression, inpatients, mental health, psychiatric nursing, violence

## Abstract

Since its development, there has been growing utilization of the Safewards package of interventions to reduce conflict and containment in acute mental health wards. The current study used the opportunity of an implementation of Safewards across one large metropolitan local health district in New South Wales Australia to evaluate change. Specific aims of the study were to measure, for the first time in Australia, changes in shift‐level reports of conflict and containment associated with Safewards introduction, and to measure any association with change in the violence prevention climate using a tool validated for use in the current study setting. Eight of eleven wards opted‐in to participating in Safewards. Implementation was conducted over a period of 24 weeks (4‐week preparation, 16‐week implementation, and 4‐week outcome phases). Conflict and containment were measured using the Patient–Staff Conflict Checklist Shift Report and violence prevention climate using the VPC‐14. From 63.2% response rate, the mean (SD) reported conflict and containment incidents per shift fell from 3.96 (6.25) and 6.81 (5.78) to 2.94 (4.22) and 5.82 (4.62), respectively. Controlling for other variables, this represented reductions of 23.0 and 12.0%, respectively. Violence prevention climate ratings did not change. Safewards was associated with significant improvements in all incidents of conflict and containment, including the most severe and restrictive types, and this was largely unaffected by outcomes measure response rate, shift or weekday/weekend reporting, or number of ward beds. Safewards is increasingly justified as one of very few interventions of choice in adult, acute mental health services and should be widely utilized.

## INTRODUCTION

Safewards is a theoretically grounded, empirically supported series of interventions for reducing conflict, for example rule breaking or aggression, and containment, for example ranging from use of patient‐requested ‘as required’ medication through to serious, restrictive practices like restraint (Bowers *et al*. 2014, 2015). Like other organized programmes to reduce conflict and containment, Safewards is needed because these incidents constitute and contribute to aggression (Papadopoulos *et al*. 2012), are associated with an unsafe and unpleasant therapeutic environment (Robinson *et al*. 2016) which, in turn, has negative ramifications for outcomes like satisfaction and therapeutic relationships (Bressington *et al*. 2011). Incidents of containment may promote disharmony, discord, and, in cases of highly coercive interventions such as restraint, are associated with significant risk of patient injury or, in extremis, death (Duxbury *et al*. 2011). In a systematic review of seclusion and restraint reduction programs in mental health settings (Goulet *et al*. 2017), Safewards was one of only two models to have been subject to a randomized trial of effectiveness, the other being the six core strategies model developed by Huckshorn (2014).

While Safewards has been successfully evaluated in a well‐conducted cluster randomized controlled trial (Bowers *et al*. 2015), further evaluation of local implementations using the most robust methodology available is advisable (World Health Organisation 2007). Evidence‐based implementations of Safewards have the potential to inform knowledge development on a number of levels (Smith & Ory 2014). At a local level, nurses, other team members, and service managers will want to know that their effort been successful, or, if not, to find out why. They offer an opportunity to teams to participate in well‐structured programmes of interventions and get involved in evaluation activity. Results will naturally be less persuasive then those from controlled trials in which comparison wards receive a resource‐equivalent alternative; nevertheless, learning can be achieved about the operationalization of the interventions themselves including their organization and delivery. Local evaluations can inform readers about the applicability of interventions when implemented in a different setting to those in the original trial, for example a number of Safewards evaluations have been conducted in forensic settings (Cabral & Carthy 2017; Price *et al*. 2016), and using outcomes measures that may be more suitable to the setting: for example, studies in forensic and non‐forensic settings have used the EssenCES (Schalast *et al*. 2008) social climate scale rather than the Ward Atmosphere Scale (Moos 1974) used by Bowers *et al*. (2015).

### Safewards intervention: literature review

Safewards is a collection of ten interventions based on an explicit mental health nursing theory (Bowers *et al*. 2014) which are intended to reduce occurrence of conflict and containment on mental health inpatient wards. The interventions are hypothesized to ameliorate potential flashpoint situations which may arise from six originating domains (the staff team, the physical environment, outside hospital, the patient community, patient characteristics, and the regulatory framework). Safewards has been thoroughly described both prior to the original trial (Bowers *et al*. 2014), in the original trial paper (Bowers *et al*. 2015) and in subsequent trials and evaluations (Baumgardt *et al*. 2019; Fletcher *et al*. 2017).

In the Safewards model and the subsequent cluster trial (Bowers *et al*. 2014, 2015), it is conflict and containment *in general*, as opposed to specific types of incident such as aggression or seclusion, that are identified as the primary outcomes for evaluation of the intervention. In any event, Bowers *et al*. (2005) point to the evidence of significant correlations between subsets of measures of more serious aggression (e.g., violence) and containment (e.g., restraint) and the total incidence of conflict and containment using broad definitions which include relatively low severity levels of conflict (verbal aggression, breaking ward rules) or, again relatively, low‐coercion containment measures (PRN medication, time out). Given this relationship, it is convenient to study all incidents as it removes the problem of high zero‐event frequency of incidents and the subsequent implications for statistical power; it is also justified since it is likely that similar interventions based on the same underlying model would be hypothesized to have the same ameliorative effect. In addition, one might also hypothesize that reduction of less serious incidents could have knock‐on benefits due to an effect of improved ward atmosphere or safety. The original Safewards trial (Bowers *et al*. 2015) demonstrated significant reductions in broadly defined conflict and containment, but the only other study thus far to examine similarly defined outcomes was that of Price *et al*. (2016) conducted in a forensic mental health service. While Price and colleagues achieved high response rate on the shift‐level data collection tool (the Patient–staff Conflict Checklist – Shift Report or PCC‐SR, Bowers *et al*. 2005), results of the non‐randomized six‐ward trial were inconclusive potentially due to poor fidelity to the Safewards intervention protocols (27.3%). Elsewhere, studies have focused on more serious incidents only, and, largely speaking, on containment measures since these are commonly recorded on centralized registers in order to audit compliance with reduction strategies. Also – and contrary to the experience of the current authors – because it has been considered too onerous a task for nurses to complete a shift‐level report for research purposes (Fletcher *et al*. 2017). Studies by Baumgardt *et al*. (2019) (outcomes: mechanical restraint, forced medication, and limitation of freedom of movement), Fletcher *et al*. (2017) (seclusion), and Stensgaard *et al*. (2019) (mechanical restraint frequency and duration, forced sedation frequency) have reported positive, but not unequivocally significant, results all based on evaluations in which good levels of intervention fidelity were attained. Bowers *et al*.'s (2015) original trial also examined a range of additional measures to identify whether Safewards' success carried over into attitudinal domains including attitudes to personality disorder questionnaire measures (Bowers & Allan 2006), self‐harm antipathy scale results (Patterson *et al*. 2007), and to a measure of ward social climate (Ward Atmosphere Scale; Moos 1974). None of these measures changed significantly over the course of the Safewards intervention.

With specific regard to the use of ward climate as an outcome, the validity of the WAS has been contested and, anyway, in Bowers *et al*.'s (2015) study was measured solely among nursing staff and not patients/consumers. In other Safewards' studies, social climate has also been investigated – both among staff and patients – with some indication using the EssenCES (Schalast *et al*. 2008) of positive change in a German study in a locked acute psychiatric setting (Baumgardt *et al*. 2019). There was also positive change using the EssenCES in Cabral and Carthy's (2017) study in a UK forensic setting, and in a study conducted in Finnish adolescent mental health inpatient units (Hottinen *et al*. 2020). Notably, the EssenCES (Schalast *et al*. 2008) was developed in forensic settings in Germany and validated in similar UK settings and has not been validated for use in a general acute setting.

### Contribution of the current study

Safewards can effectively reduce conflict and containment in acute mental health wards (Bowers *et al*. 2015). In this study, we examined whether such gains were mirrored in a 'real life' scenario outside the context of a controlled trial. We also aimed to examine the use of outcomes related to serious conflict and highly coercive containment as well as broader measures of those outcomes for the first time in a study in Australia. Finally, we aimed to measure one aspect of social climate, the violence prevention climate, using a tool that was validated during the course of the study (Dickens *et al*. In Press) and among both staff and patients. The specific hypotheses of the study were that introduction of Safewards would be associated with significant reductions in reported (i) conflict; (ii) serious conflict (physical violence); (iii) containment; (iv) highly coercive containment (seclusion, restraint, forced IM) after controlling for potential confounding variables; and (v) with significant improvements in the measured violence prevention climate.

## METHODS

### Design

The study used a longitudinal pre‐/post‐test design. Each participating unit received a similar level of support to implement the intervention across a 4‐week baseline/preparatory phase, a 12‐week implementation phase, and a 4‐week outcome phase. Introduction was stepped across the service such that units commenced the baseline phase at 1–2 week intervals so that no more than three wards at any one time were in the preparatory phase. Organization and delivery of the intervention were undertaken by a single full‐time project officer, an experienced registered nurse with a Master's degree in mental health nursing (TT) under the supervision of a professor of mental health nursing (GLD). Additionally, the project was informed by a steering group comprising five core additional members (two consumer representatives, one consumer representative manager, one clinical nurse consultant, and one executive nurse manager) plus others who advised and attended group meetings when available or when required. The role of the Steering Group was to advise on all aspects of implementation and evaluation.

### Participants and setting

The study was conducted in one large metropolitan local health district in Sydney NSW (population served approximately one million). Invitations to express an interest in participation were sent to managers of all adult (*n* = 10) and adolescent (*n* = 1) inpatient mental health units in the district. Full information was provided and all nurse unit managers provided with an opportunity to meet and ask questions of the research team before responding. As a result, *n* = 9 units expressed an interest to participate. Those choosing not to participate were an inpatient adolescent inpatient mental health unit (reported that they currently utilized Safewards interventions) and an adult psychiatric high dependency unit (reported they had formally introduced the Safewards interventions over the past two years). Safewards was introduced to nursing staff via hour long ward in‐service sessions on those wards who expressed an interest in participation. At this point, one unit manager ceased responding to repeated requests for us to attend their ward for this purpose and the ward was excluded from the study. Thus, the Safewards intervention was commenced on eight wards (total beds = 142). In terms of outcome measures, potential participants were, for the violence prevention climate scale (Hallett *et al*. 2018), any staff member or patient resident on the unit during the baseline or outcome phases of the study. The PCC‐SR (Bowers *et al*. 2005) was completed routinely across the service during the study period, and individual staff consent was not sought for use of these data in the analysis.

### Intervention

Safewards was thoroughly described prior to the original trial (Bowers *et al*. 2014), in the original trial paper (Bowers *et al*. 2015), and in descriptions of subsequent trials and evaluations (Baumgardt *et al*. 2019; Fletcher *et al*. 2017). Safewards is a collection of 10 interventions based on an explicit mental health nursing theory (Bowers *et al*. 2014) which are intended to reduce occurrence of conflict and containment on inpatient wards. The interventions are hypothesized to ameliorate potential flashpoint situations which may arise from six originating domains (the staff team, the physical environment, outside hospital, the patient community, patient characteristics, and the regulatory framework).

A plan for Safewards implementation was devised with a group of select staff from each participating ward who volunteered or were nominated by the unit manager to facilitate the application of the interventions on their unit. Interventions were contextualized to each ward and introduced incrementally over the 12‐week implementation phase with the support of the project officer. Order of implementation of interventions was decided by participating wards. Five wards decided to implement all 10 interventions while two wards chose to implement 9 interventions and one ward 8 interventions. Support provided during the preparation and implementation phases of the study included 'train‐the‐trainer' sessions for clinical nurse consultants and educators, introductory in‐service education sessions, regular unit visits and telephone calls from the project officer, extensive intranet‐based educational resources including those from Safewards studies in the UK and Victoria, Australia. All materials such as sensory boxes were funded from project resources, sourced and constructed by the project officer. In addition, further props to support implementations such as discharge messages and soft words were devised and delivered throughout the support phase of the project.

### Measures

#### Patient–staff Conflict Checklist Shift Report (PCC‐SR; Bowers et al. 2005)

Numbers of incidents of conflict (e.g., aggression, rule breaking) and containment (e.g., seclusion/restraint) were extracted from shift reports on the Patient–Staff Conflict Checklist. This is a tool developed by Bowers *et al*. (2005) and used routinely on the wards under study during the current investigation. In this study, internal reliability for conflict items was acceptable (Cronbach’s α = 0.730). Internal reliability for types of containment incident would not be expected since each is known to have its own unique profile in terms of overall acceptability for management of both aggressive and self‐harming behaviours (Bowers *et al*. 2007; Hosie & Dickens 2018).

#### VPC‐14 (Hallett et al. 2018)

This is a 14‐item scale based on extensive systematic and conceptual literature reviews of the perceptions of staff and patients regarding violence prevention in mental health settings (Hallett & Dickens 2017; Hallett *et al*. 2014), and on empirical studies (Hallett & Dickens 2015; Hallett *et al*. 2016). In its initial UK validation study, it was found to have good psychometric properties including internal reliability, convergent validity, and test–retest reliability. The scale comprises a two‐factor structure namely ‘patient actions’ (things patients do that prevent violence) and ‘staff actions’ (the things that staff do). For the current study, the tool was reviewed by the Safewards’ project steering committee. While numerous suggestions for amendments were made, it was understood that the current items were based on extensive research and changes should be as limited as possible. As a result, just one amendment was made to item 3: ‘Staff on the ward are good at talking down aggressive patients’. This was viewed as lacking in clarity; specifically, the term 'talking down' (intended to mean ‘de‐escalate’) was taken to mean 'talking down to' (i.e., ‘in a condescending manner’). Therefore, we added a footnote informing respondents that 'talking down' was intended to mean 'in a way that intends to calm people down or de‐escalate them'. Items are rated on a 5‐point Likert scale (strongly agree to strongly disagree). In the current study, the internal reliability of the scale was good (Cronbach’s α = 0.838).

#### Fidelity checklist

The 10‐item fidelity checklist was used to determine whether there was visible evidence that staff on each ward had implemented each intervention. This was the same tool used in the original Safewards trial (Bowers *et al*. 2015) and comprised 1‐item per intervention. An example item for the 'soft words' intervention: 'Is an appropriate message of the day displayed in a prominent place on the ward?'. All items are scored Yes/No with to derive an overall total score (maximum 10).

#### Ward characteristics

The following ward characteristics were collected to assist with analysis: number of beds, ward function (acute versus non‐acute service).

### Procedure

The study, including all procedures described here, was granted ethical approval by the South Western Sydney Local Health District Human Research Ethics Committee (Ref: 2019/ETH10615). Data were collected between April 2019 and January 2020. The PCC‐SR had been introduced as a mandatory routine outcome measure on all units in the LHD prior to study commencement and ethical approval covered the use of de‐identified versions of this to evaluate the Safewards intervention and consent was not taken from individual nurses to use these data. The PCC‐SR is completed by the nurse‐in‐charge on each shift, and a copy was forwarded to the Safewards project co‐ordinator before the end of the shift. Wards were supported with data collection via the production and circulation of a weekly email provided to each NUM detailing the response rate. Where response rate fell below a pre‐determined level (70%), NUMs were provided feedback and offered suggestions and help to achieve better response rates.

The VPC‐14 was circulated in the baseline and outcome study phases for completion by both staff and patients. Very minimal demographic data were collected (staff or patient and ward name), and consent was taken to be implied by return of the completed questionnaire. For staff, the nurse unit manager was provided with sufficient copies of the VPC‐14 and participant information sheets for circulation to staff. For patients, information sheets were circulated and one of the research team attended the ward on several occasions to follow‐up those who expressed an interest and to offer any assistance required to complete the form. Because identifying details were not collected, we could not determine whether any individuals who completed the measure at both baseline and outcome had done so on both iterations.

The fidelity checklist was completed by the project officer on a minimum of two occasions (Mdn = 2, range 2–3) with one check always occurring in the outcome phase of the study.

### Data analysis

Similar to Bowers *et al*. (2015), the primary outcomes for the study were counts of conflict and containment. Given the excess of completed PCCs with zero counts (35.6% for conflict, 10.4% for containment), we adopted a similar strategy of a two‐part hurdle model. Part one, the hurdle, involves examination of zero‐count reports, that is the frequency of shifts on which the count of either conflict or containment was nil. Examination at this level involves analysis of whether a significant change occurs in the frequency of zero‐count shifts over the course of the study period. The second part of the model examines the count of conflict and containment events using a negative binomial model which allows for over‐dispersion (variance greater than the mean) resulting from excessive zero‐count data. It is a generalization of a Poisson model, but compared with Poisson regression confidence intervals are likely to be narrower. The general linear model function in SPSS was used to model the relative risk of conflict and containment events across the study phases when accounting for potential covariates. Variables entered into the model were study phase (baseline, implementation, and outcomes), shift (morning, afternoon, and night), weekday vs. weekend, number of beds (expressed as the relative change in risk per 5‐bed increase), and PCC‐SR response rate (expressed as the relative change in risk per 10% change in response rate). Analyses were run separately for total conflict and total containment and separately also for serious conflict in the form of physical aggression (proportion of zero‐event shifts and counts of incidents of physical aggression across the period) and for highly coercive containment in the form of restraint, seclusion, or forced medication.

### Role of the funding source

The funder had no role in design, conduct of the intervention, data collection, data analysis, or writing of the study report.

## RESULTS

The total number of completed PCCs returned to the project officer over the 20‐week evaluation period was 2124 /3360 (Response rate 63.2%). Missing data were not distributed evenly. In terms of ward, (Χ^2^ = 912.28 *P* < 0.001) inspection of standardized residuals indicated there were significantly more missing data from three wards (Mdn = 31.9% complete data, range 20.0–43.6%) and significantly less on the remaining five (Mdn = 78.8% complete data, range 63.2–90.5%). This was, in effect, a function of study site since all five wards with the highest response rates were located on one campus and the three wards with the lowest response rates at two other sites. There were significantly fewer missing outcome data from PM shifts (374/1125; 33.2% missing) compared with AM (425/1117; 38.1% missing) and night (423/1098; 38.5% missing) (Χ^2^ = 8.22, df = 0.2 *P* = 0.016; standardized residual for PM shift = −1.9), though this was fairly marginal. There were fewer missing data in the baseline phase (28.1%; 189/672) than the implementation phase (37.9%; 763/2016) and the outcome phase (42.7%; 287/672; Χ^2^ = 32.75 df = 2 *P* <0.001 standardized residual for baseline phase = −3.7, and for outcome phase = 2.5 (i.e., missing data significantly underrepresented at baseline and overrepresented at outcome).

### Fidelity to Safewards interventions implementation

By the final fidelity audit on each ward, overall fidelity was 73.7%, that is across wards seven interventions were being adequately implemented (range 4–9). The number of participating wards was too small to conduct correlational analyses to determine whether the number of correctly implemented interventions was associated with changes in conflict or containment. Visual inspection of rankings revealed no obvious pattern.

### Incidence of conflict and containment across implementation

Mean conflict and containment incidents for the entire period of study are presented in Table 1. Correlations between the conflict (minus the serious aggression count) and the serious aggression count (*r* = .539, *P* < 0.001) and between containment (minus the highly coercive containment count) and the highly coercive containment (*r* = 0.348, *P *< 0.001) were positive and significant.

**Table 1:** Unadjusted conflict and containment rates across implementation

	M (SD) [Event rate]	Mdn (IQR)	Risk of events (*n*/N) [Hurdle rate]
*PCC Conflict*			
Any conflict (Count)	3.33 (5.11)	2.0 (0–4)	–
Any (Any conflict event/shift)	–	–	0.64 (1366/2124)
Physical aggression (Count)	0.14 (0.35)	0.0 (0–0)	–
Physical aggression (Any event/shift)	–	–	0.14 (304/2124)
*PCC Containment*			
Any containment (Count)	5.91 (5.00)	5.0 (2–9)	–
Any (Any containment event/shift)			0.90 (1900/2124)
Highly coercive containment (Count)	0.13 (0.51)	0 (0–0)	
Highly coercive containment (Any event/shift)	–	–	0.083 (176/2124)

#### Shift

There were significantly more conflict incidents reported for AM shifts (M[SE] (5.22 [0.21]) than for PM (3.56[0.20]) or night (1.74[0.21]) shifts. The difference between PM and night shifts was also significant. There were more incidents of physical aggression reported for AM (0.44[0.04]) than for PM (0.35[0.04]) or night shifts (0.08[0.04]). The difference between PM and night shifts was also significant. Differences between number of containment incidents were only significant between AM (6.84[0.21]) and night (5.83[0.21]) shifts. Highly coercive containment incidents (seclusion, restraint, forced IM) were more frequently reported for AM (0.19[0.02]) than for night shifts ((0.06[0.02]) but not than for PM shifts; however, they were more frequently reported for PM (0.14[0.02]) than for night shifts. Relative risk ratios for the count of incidents per shift are presented in Tables 2 and 3.

**Table 2:** Relative risk of conflict and containment events

	M (SD)	Rate ratio (95% CI)		*P*
		Crude	Adjusted	
Conflict				
Study Phase				
Baseline	4.0 (6.2)	1.0 (ref)	1.0 (ref)	
Implementation	3.2 (4.9)	0.80 (0.71–0.91)	0.83 (0.74–0.93)	0.002
Outcome	2.9 (4.2)	0.73 (0.62–0.85)	0.77 (0.66–0.89)	0.001
No. of beds (per 5 increase)		1.27 (0.9–1.81)	1.19 (0.84–1.68)	0.330
Week day versus weekend		0.98 (0.87–1.09)	0.97 (0.87–1.07)	0.547
Shift				
Night		1.0 (ref)	1.0 (ref)	
Morning		0.77 (0.69–0.86)	0.78 (0.70–0.87)	<0.001
Afternoon		0.39 (0.35–0.44)	0.40 (0.35–0.45)	<0.001
Ward response rate (per 10% increase)		0.91 (0.74–1.13)	0.94 (0.77–1.15)	0.5478
Containment				
Study Phase				
Baseline	6.8 (5.8)	1.0 (ref)	1.0 (ref)	
Implementation	5.9 (4.7)	0.87 (0.82–0.91)	0.87 (0.82–0.91)	0.002
Outcome	5.8 (4.6)	0.88 (0.82–0.94)	0.88 (0.82–0.94)	0.001
No. of beds (per 5 increase)		1.23 (0.88–1.73)	1.19 (0.85–1.65)	0.308
Week day versus weekend		1.05 (1.00–1.10)	1.04 (0.99–1.09)	0.080
Shift				
Night		1.0 (ref)	1.0 (ref)	
Morning		0.99 (0.94–1.04)	0.78 (0.70–0.87)	0.795
Afternoon		0.94 (0.89–0.99)	0.40 (0.35–0.45)	0.045
Ward response rate (per 10% increase)		0.91 (0.74–1.13)	0.94 (0.77–1.15)	0.371

**Table 3:** Relative risk of serious conflict and highly coercive containment events

	M (SD)	Rate Ratio (95% CI)		*P*
		Crude	Adjusted	
Serious Conflict (Physical aggression)				
Study Phase				
Baseline	4.0 (6.2)	1.0 (ref)	1.0 (ref)	
Implementation	3.2 (4.9)	0.75 (0.55–1.02)	0.82 (0.74–0.90)	0.002
Outcome	2.9 (4.2)	0.62 (0.41–0.94)	0.65 (0.59–0.72)	<0.001
No. of beds (per 5 increase)		1.23 (0.85–1.77)	1.14 (1.03–1.26)	0.001
Week day versus weekend		1.13 (0.85–1.49)	1.19 (1.08–1.31)	<0.001
Shift				
Night		1.0 (ref)	1.0 (ref)	
Morning		0.89 (0.67–1.17)	0.92 (0.83–1.02)	0.070
Afternoon		0.14 (0.10–0.22)	0.15 (0.14–0.16)	<0.001
Ward response rate (per 10% increase)		0.89 (0.72–1.10)	0.92 (0.83–1.02)	0.067
Containment (Highly coercive)				
Study Phase				
Baseline	6.8 (5.8)	1.0 (ref)	1.0 (ref)	
Implementation	5.9 (4.7)	0.70 (0.49–1.02)	0.70 (0.49–1.01)	0.058
Outcome	5.8 (4.6)	0.27 (0.15–0.49)	0.26 (0.14–0.47)	<0.001
No. of beds (per 5 increase)		1.56 (0.89–2.71)	1.39 (0.85–2.29)	0.188
Week day versus weekend		0.83 (0.57–1.21–1.10)	0.81 (0.56–1.18)	0.276
Shift				
Night		1.0 (ref)	1.0 (ref)	
Morning		1.03 (0.71–1.52)	1.09 (0.75–1.59)	0.661
Afternoon		0.48 (0.31–0.76)	0.50 (0.32–0.78)	0.002
Ward response rate (per 10% increase)		0.75 (0.55–1.02)	0.80 (0.61–1.06)	0.122

#### *Weekdays* versus*Weekends*

No significant differences were found between the mean number of incidents of conflict or containment reported between weekends and weekdays (see Tables 2 and 3).

#### Ward

Individual wards are denoted as either A (acute ward 1–4) or NA (non‐acute ward 1–4). Conflict incidents were most commonly reported on A3 (M [SD] (9.10 [7.57]), A1 (5.83 [5.05]), and A2 (4.61 [5.72]) and least frequently on NA4 (0.91 [1.61]), NA2 (1.02 [1.57]), and A4 (1.49 [1.83]). A3 had significantly more incidents than every other ward. A1 had significantly more conflict incidents than every other wards except, of course, A3, but also A2 which, in turn, had significantly more incidents than all wards apart from NA3. Four wards all with similar rates (NA1, NA2, NA3, and A4) had significantly fewer incidents than all other wards. Containment incidents were most frequent on A3 (11.77 [4.76]), A1 (10.37 [4.25]), A2 (9.79 [3.62]), and A4 [8.18 [3.25]). A3 had significantly more containment episodes than all other wards; A1 had significantly more containment episodes than all other wards except A3 and A2, and A4 had significantly more episodes than the four non‐acute wards. Least containment episodes were on NA3 (1.48 [1.57]) and NA4 (1.86 [1.50]), and these two wards had significantly fewer reported events than all other wards. Thus, conflict and containment rates largely mirrored the acute/non‐acute ward function with the exception of A4 where conflict rates were similar to non‐acute wards but containment rates significantly higher.

#### Change across implementation

Table 2 shows raw mean (SD) rates of conflict and containment incidents and Table 3 equivalent figures for more serious conflict and coercive containment incidents reported per completed PCC across the three phases of the study. For conflict, the mean rate fell significantly from baseline phase to implementation phase and from baseline phase to outcome phase but did not fall further from implementation phase to outcome phase. For containment, the mean rate fell significantly from baseline phase to implementation phase and from baseline phase to outcome phase, but, again, there was no significant change between implementation and outcome phases. There was no significant difference in conflict‐free event days across the three study phases. There were more containment free days in the baseline period (70/413 14.5%) than in the outcome phase (18/367 4.9%) though there were more missing data in the outcome phase (see above).

Tables 2 and 3 also show the relative risk of conflict and containment (Table 2) and more serious conflict and containment issues (Table 3) for study phase (reference category: baseline), N beds, weekday versus weekend, shift (reference category: night), and ward PCC response rate. For all incidents (Table 2), both crude and adjusted rates are similar across analyses suggesting results are robust and without major confounds. Relative to baseline, the risk of conflict had reduced 23% by the study follow‐up period and that of containment had reduced by 12% after controlling for other important factors.

Similarly, for aggression and highly coercive containment (Table 3) crude and adjusted odds are similar suggesting robust results. For physically aggressive incidents, the risk at outcome phase relative to implementation phase had fallen by 35.0%, and for highly coercive containment, the equivalent figure was 74.0%. Serious physical aggression was also significantly higher on wards with more beds and on weekends compared with weekdays.

## DISCUSSION

We facilitated implementation of the Safewards package of interventions across eight adult mental health wards in a large local health district in metropolitan NSW. We evaluated the implementation by examining routinely reported rates of conflict and containment reported across a 20‐week period comprising baseline (4‐w), implementation (12‐w), and outcome (4‐w). In addition to change in overall rates of conflict and containment, controlling for response rate, shift, day of week, and ward size (beds) we also examined rates of the most serious conflict and most coercive containment events. Finally, we examined whether implementation of Safewards was associated with changes in a measure of the violence prevention climate using a tool validated during the course of the study. There was no control group and no randomization. After adjusting for covariates, overall conflict reduced by 23.0% relative to baseline data while containment fell by 12.0%. For more serious conflict, reduction was in the order of 35.0% and for highly coercive containment 74.0%. The violence prevention climate measured by the VPC‐14 did not change significantly across the implementation period. Outcome measures were taken in the context of good fidelity to the Safewards interventions. Overall reductions in conflict and containment were somewhat similar in magnitude (15.0% and 26.4%) to those reported by Bowers *et al*. (2015) in their cluster randomized controlled trial using the same outcome measure and were somewhat better than those achieved by Price et al. (2017) in their non‐randomized trial in a forensic setting where reported intervention fidelity was relatively poor. At face value, therefore, the current study amplifies findings that Safewards is an effective intervention in acute mental health settings for reduction of conflict and containment and seems to suggest, as one would expect, that fidelity to the interventions selected plays an important role in success. The additional analyses of more serious conflict behaviours in the form of physical aggression and of more highly coercive containment measures suggest that particular benefit is derived from Safewards. While they were relatively rare events occurring at rates of 0.14 and 0.13 events per shift, respectively, they did occur on 14.0% and 8.3% of all recorded shifts.

One major contribution of the current study is in its exposition of how the positive results were achieved in terms of implementation. This will allow other services to judge whether they can aim to match or even better our results. In brief, we used study funding to second one experienced Masters‐level prepared mental health nurse to a full‐time position for one calendar year. Within that year, ethical approval for evaluation was sought and gained, necessary links made with services, interventions and required resources identified, planned, and delivered. A steering group comprising individuals with relevant expertise was initiated and met regularly throughout the project to provide guidance. As described in this paper, Safewards was delivered on eight of eleven wards within the Local Health District. A further achievement, unique thus far in the Australian context, was the delivery of data from shift‐level reports about conflict and containment incidents and not solely incidents of seclusion (Fletcher 2017). While overall response rate for the PCC‐SR was not perfect (63.2%), it was higher than that achieved (around 53.0%) in the original Safewards study (Bowers *et al*. 2015). The explanation for the relatively good response rate must be, as in a study by Price *et al*. (2016), that the tool was mandated for use as a routine service‐wide outcome measure and not subject to research consent as in the original Safewards trial. The very high rates of 80.0% plus achieved on some wards suggested that this sort of routine data is achievable where unit managers are onboard and committed to assisting the project. One of our key recommendations for the current service was to maintain the PCC‐SR as a routine outcome measure for nursing across the services. Finally, we note that Safewards was not implemented in precisely the same way as in the original trial where a baseline period of 8 weeks was followed by supported and unsupported periods of a similar length. The literature thus far suggests some variation in how to implement the intervention and service managers are advised to scrutinize the emerging accounts (Baumgardt *et al*. 2019; Fletcher *et al*. 2019; James *et al*. 2017; Price *et al*. 2016; Stensgaard *et al*. 2019) to inform their selection of which best suits their needs.

### Limitations

The sample size (number of participating wards) was too small to allow detailed analysis of the effect of potential mediating variables such as implementation fidelity. In any event, the measure of fidelity used is somewhat crude and reflects largely visually obvious signs of implementation and not of more encultured team responses. Negative binomial and Poisson regression differ, for example the former is associated with narrower confidence interval estimates, but there is no general answer as to which is preferable (Ver Hoef & Boveng 2007). We selected negative binomial modelling as our a priori strategy rather than choosing to make multiple analyses of the data to select the best‐fitting model. Any causal inference about the effectiveness of Safewards in reducing conflict and containment cannot be drawn from a study using a before‐after design; without control wards, randomization, and blinding – as present in the original Safewards trial – any contribution made by the study intervention could be inflated simply by chance, observer effects, or other initiatves and developments in the study setting. Nevertheless, our approach to capturing valid data lends some rigour to our findings and adds to the growing evidence base for the intervention in acute mental health services internationally. The time‐limited nature of the study meant that we could not capture sustainability of intervention effectiveness over a longer period. While we have not yet conducted a cost‐benefit analysis, we would anticipate that some of the cost of continuing such a role would be offset by savings made by conflict and containment‐related gains.

## CONCLUSIONS AND RECOMMENDATIONS

Safewards is an increasingly evidence‐based intervention for reduction of conflict and containment in acute mental health services. Services who choose not to use the intervention, or to use an alternative with less robust supporting evidence, are in danger of acting against the existing and emerging evidence. Routine outcome measures of conflict and containment at shift level provide valid data for intervention evaluation and should be utilized extensively.

## RELEVANCE FOR CLINICAL PRACTICE

Safewards is currently one of the best‐evidenced nursing interventions for containment and conflict reduction. The current study demonstrates that Safewards can be implemented with positive results in a time‐limited period across multiple units. High quality, relevant outcome data can be sourced from routinely gathered measures. The interventions need not be implemented inflexibly. Collection of social climate data such as the VPC‐14 used here could also be incorporated as a routine measure to monitor ongoing initiatives.
